# Empagliflozin in children with glycogen storage disease-associated inflammatory bowel disease: a prospective, single-arm, open-label clinical trial

**DOI:** 10.1038/s41598-024-59320-z

**Published:** 2024-04-15

**Authors:** Zhiling Li, Xiaoyan Zhang, Huan Chen, Hanshi Zeng, Jiaxing Wu, Ying Wang, Ni Ma, Jiaoli Lan, Yuxin Zhang, Huilin Niu, Lei Shang, Xun Jiang, Min Yang

**Affiliations:** 1 Department of Pediatrics, Guangdong Provincial People’s Hospital, Guangdong Academy of Medical Sciences, Southern Medical University, Guangzhou, China; 2 Department of Gastroenterology, Guangzhou Women and Children’s Medical Center, Guangzhou Medical University, Guangzhou, China; 3 Department of Pathology, Guangdong Provincial People’s Hospital, Guangdong Academy of Medical Sciences, Southern Medical University, Guangzhou, China; 4 Department of Health Statistics, School of Public Health, Fourth Military Medical University, Xi’an, China; 5 Department of Pediatrics, The Second Affiliated Hospital, Fourth Military Medical University, Xi’an, China

**Keywords:** Empagliflozin, Glycogen storage disease, Inflammatory bowel disease, Clinical trial, Children, Diseases, Endocrinology, Gastroenterology, Medical research

## Abstract

Glycogen storage disease type Ib (GSD-Ib) is a rare inborn error of glycogen metabolism caused by mutations in SLC37A4. Patients with GSD-Ib are at high risk of developing inflammatory bowel disease (IBD). We evaluated the efficacy of empagliflozin, a renal sodium‒glucose cotransporter protein 2 (SGLT2) inhibitor, on colonic mucosal healing in patients with GSD-associated IBD. A prospective, single-arm, open-label clinical trial enrolled eight patients with GSD-associated IBD from Guangdong Provincial People's Hospital in China from July 1, 2022 through December 31, 2023. Eight patients were enrolled with a mean age of 10.34 ± 2.61 years. Four male and four female. The endoscopic features included deep and large circular ulcers, inflammatory hyperplasia, obstruction and stenosis. The SES-CD score significantly decreased at week 48 compared with before empagliflozin. Six patients completed 48 weeks of empagliflozin therapy and endoscopy showed significant improvement or healing of mucosal ulcers, inflammatory hyperplasia, stenosis, and obstruction. One patient had severe sweating that required rehydration and developed a urinary tract infection. No serious or life-threatening adverse events. This study suggested that empagliflozin may promote colonic mucosal healing and reduce hyperplasia, stenosis, and obstruction in children with GSD-associated IBD.

## Introduction

Glycogen storage disease type Ib (GSD-Ib) is an autosomal recessive genetic disease caused by a defect in the SLC37A4 gene encoding the glucose-6-phosphate transporter (G6PT)^1–3^. It is characterized by hypoglycaemia, excessive accumulation of liver and kidney glycogen, neutropenia, neutrophil dysfunction, and high susceptibility to various infectious diseases, including recurrent bacterial infections, oral and perianal ulcerations, bloody diarrhoea, abdominal pains, and histological inflammation of the intestinal mucosa; up to 80% of patients with GSD-Ib have evidence of inflammatory bowel disease (IBD)^4–7^. IBD was first reported in patients with glycogen storage disease type Ib nearly 40 years ago^8^. The disease has been described as “Crohn's-like colitis”^9^ or “IBD-like colitis”^10,11^. We previously reported that 16 of 18 patients with GSD-Ib and neutropenia were diagnosed with IBD and 2 with colitis on the first endoscopy^12^. These results support the notion that there is a causal relationship between neutropenia and/or neutrophil dysfunction and IBD in patients with GSD-Ib^10,13^. We refer to this disorder as GSD-associated IBD.

Neutropenia is an essential finding in GSD-Ib and GSD-associated IBD; in addition to diet management and cornstarch maintenance of blood glucose, 5-aminosalicylic acid and granulocyte-colony stimulating factor (G-CSF) are commonly used for the management of neutropenia-induced infections and colitis in patients with GSD-Ib^10,14,15^. However, G-CSF has not shown sufficient efficacy for the prevention and treatment of GSD-associated IBD, and therapeutic options for patients who do not respond to traditional therapies have been limited owing to the adverse effects of glucocorticoids and immunomodulators on this metabolic disorder. Case reports have shown that biologics such as infliximab or adalimumab can successfully relieve clinical symptoms^16,17^; however, our previous experiment showed that infliximab did not improve mucosal healing in patients with GSD-associated IBD.

Recently, an alternative treatment option to G-CSF, such as empagliflozin, an inhibitor of kidney sodium-glucose cotransporter 2 (SGLT2), which restores normal neutrophil counts by reducing the level of 1,5AG6P in the blood, has been preferred ^18^. There is growing evidence that empagliflozin exerts a positive effect by increasing the number and function of neutrophils in patients with GSD-Ib, which can improve clinical symptoms, reduce the paediatric Crohn’s disease activity index (PCDAI) score, and improve the quality of daily life of GSD-Ib patients with IBD^19–21^. However, remission measured with clinical indicators does not correlate with endoscopic remission in adults or children^22^, and whether empagliflozin promotes endoscopic remission and mucosal healing has not been reported. In this study, we determined the efficacy and safety of empagliflozin in children with GSD-associated IBD after 48 weeks by evaluating clinical symptoms, laboratory indicators, the PCDAI, and especially the endoscopic features and histological activity, which may provide an important clinical strategy for patients with GSD-associated IBD.

## Methods

### Study design and participants

A prospective, single-centre, single-arm, open-label clinical trial was conducted between July 5, 2021, and December 31, 2023, at Guangdong Provincial People's Hospital in China. Before trial initiation, the protocol, consent form, and patient information sheet were reviewed and approved by the Medical Ethics Committee of Guangdong Provincial People’s Hospital (ID: 202205306). The trial was conducted according to the principles of the Declaration of Helsinki and the International Conference on Harmonisation Good Clinical Practice guidelines. Informed consent was obtained from all participants or their legal guardians (for participants under 18 years of age). This study was retrospective registered in Chinese Clinical Trials Registry (ChiCTR2400080773) (06/02/2024).

Eligible patients were male or female and 1–18 years in age at the time of signing informed consent. GSD-Ib was confirmed by genetic testing, and IBD was confirmed by endoscopy. In addition, their vital signs were stable. All included patients volunteered to participate in the trial, and informed consent forms were signed by the patients and their legal guardians. Among the key exclusion criteria were the presence or history of allergies to empagliflozin; unstable vital signs; insufficiency or failure of the heart, liver, kidney, or other organs; active tuberculosis; malignant tumours; and any disease or condition that may have affected the conduct of the study. Patients who had participated in other studies or who were not considered suitable for inclusion by the investigator were excluded.

Disease activity was measured using the PCDAI and weighted pediatric Crohn’s disease activity index (wPCDAI)^23^. The clinical response (moderate/severe disease improvement to mild/inactive disease) to PCDAI was best reflected by a ≥ 12.5 decrease in the PCDAI score, and a PCDAI score of < 10 best reflected inactive disease^23,24^. The wPCDAI score < 12.5 is remission, > 40 is moderate activity, > 57.5 is severe activity, and a score > 17.5 before and after treatment is a small improvement, and > 37.5 is a moderate improvement^23^. Endoscopic and histological activity was assessed according to the SES-CD score^25^ and Geboes method^26^.

### Procedures

**Figure 1:**
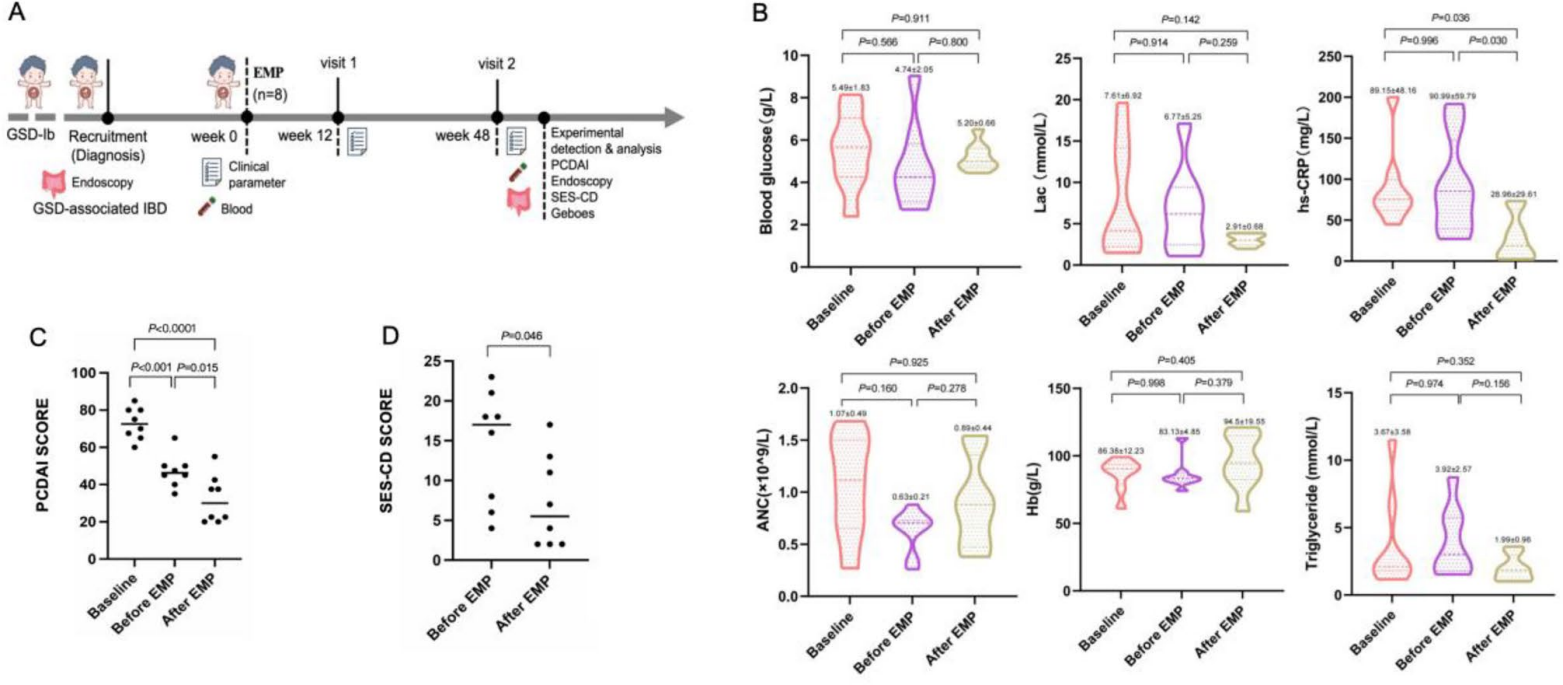
Cohort characteristics and clinical indices. (**A**) Flow chart of the clinical trials. (**B**) Compared with those at baseline and before empagliflozin treatment, blood glucose levels were more stable; lactate and hs-CRP levels were decreased, and ANC, haemoglobin, and triglyceride levels were improved. (**C**) The PCDAI scores were significantly lower at week 48 than at baseline and before treatment with empagliflozin (P < 0.001 and P = 0.015). (**D**) The SES-CD scores at week 48 were significantly lower than before treatment with empagliflozin (P = 0.045) .

Clinical data were collected and followed up by the investigators according to the study protocol (Fig. 1A). Body weight and height were recorded to calculate BMI. Data on clinical symptoms; physical examination results; vital signs; laboratory indicators including the erythrocyte sedimentation rate (ESR), high-sensitivity C-reactive protein (hs-CRP) level, white blood cell count, absolute neutrophil count (ANC); platelet count; and levels of haemoglobin, blood glucose, lactate, albumin, uric acid, cholesterol, and triglycerides were recorded.

Patients received empagliflozin 0.25 ~ 0.50 mg/kg/day for 48 weeks. The standard diet was as follows: 2 g/kg uncooked corn starch (UCCS), Q6h, as well as a lactose-free formula, polymer formula, or elemental formula to maintain blood glucose. Telephone follow-up at 12-week intervals including whether the drug was taken on time as prescribed and adverse drug reactions, and endoscopic and histological examinations were conducted in the Department of Pediatrics, Guangdong Provincial People's Hospital, after 48 weeks. At the follow-up visit, discontinuation criteria were evaluated; diaries were collected, reviewed, and transcribed; and diet and lifestyle advice were provided. An end-of-trial follow-up visit for safety assessments was scheduled 12 weeks after the end of treatment. Patients who prematurely discontinued treatment had a follow-up visit scheduled for 48 weeks.

Baseline endoscopy was performed either in the first 12 months or before the clinical trial, and all patients who completed treatment had an endoscopy and biopsy after 48 weeks. The extent of the mucosal injury was assessed by two gastroenterologists and endoscopists according to the SES-CD score and histologically assessed by the same pathologist according to the Geboes criteria.

### Outcomes

The primary endpoint was patients whose colon mucosal ulceration, inflammatory hyperplasia, and stenosis and obstruction were alleviated by endoscopy. Primary endpoints were prospectively evaluated by gastroenterologists and endoscopists.

The secondary endpoints were the PCDAI score; laboratory test results; ESR; hs-CRP; white blood cell count; ANC; platelet count; haemoglobin, blood glucose, lactate, albumin, uric acid, cholesterol, and triglyceride levels; and the number of treatment-emergent adverse events.

### Safety and clinical adverse events

All possible side effects, including but not limited to allergies/anaphylaxis, grade 3 hypoglycaemia, oral fungal infection, genital fungal infection, urinary tract infection, rash, itching, ketoacidosis, lactic acidosis, and dehydration, were recorded^19^ as observed by the investigator or reported by the patient. The disease severity (mild, moderate, severe), duration, outcome, and possible relationship with the study treatment were assessed.

### Statistical analysis

All statistical analyses were performed with SAS (version 9.4). Descriptive statistical analysis was used; quantitative variables are expressed herein as the mean ± standard deviation (SD), and categorical variables are expressed as frequencies. Differences between clinical quantitative indices among groups were tested by using one-way analysis of variance (ANOVA) with Bonferroni correction.

### Ethical approval

This study was approved by the Medical Ethics Committee of Guangdong Provincial People’s Hospital (ID: 202205306).

## Results

### Patient demographics and baseline characteristics

Eight patients were enrolled in this study, six patients (patients 1, 2, 3, 4, 5, and 7) were prescribed empagliflozin for 48 weeks at a dose of 0.25–0.50 mg/kg/d, two patients (patients 6 and 8) received intermittent empagliflozin of 0.20–0.22 mg/kg/d for a total duration of less than 8 weeks. All eight patients were received endoscopy and biopsy at 48 weeks. The data reported here are for patients enrolled between July 1, 2022, and November 14, 2023, from the time they underwent endoscopy within 12 months before enrolment to the time after empagliflozin treatment, with a cut-off date of December 31, 2023. Of the 8 patients, 4 were female and 4 were male. The mean age at diagnosis for GSD-Ib patients was 0.88 years (range 0.4–2.3 years), the age at enrolment was 10.34 years (range 4.5–12.5 years), and the mean BMI was 16.81 ± 2.59 kg/m^2^ (range 14.48–22.32 kg/m^2^) (Table 1).

**Table 1:** Baseline characteristics of the patients.

	Patient 1	Patient 2	Patient 3	Patient 4	Patient 5	Patient 6	Patient 7	Patient 8	Mean	SD
Sex	Male	Male	Female	Female	Male	Female	Male	Female		
Age at diagnosed, year	0.67	1.5	0.5	0.6	0.4	0.5	0.6	2.3	0.88	0.67
Age of the first endoscopy, year	5.3	8.2	9.8	7.8	2.6	9	8.8	9.6	7.64	2.48
Enrolment age, year	9.2	12.5	11.6	10.1	4.5	11	11.3	12.5	10.34	2.61
Body weight, kg	18	20	25	26	20	45	30	23.5	25.94	8.62
BMI, kg/m^2^	15.15	15.39	14.08	17.35	18.14	22.31	16.52	17.46	17.04	2.52
UCCS*, g/kg/Day	10.7	8.1	9.1	11.9	5.3	7.7	11.3	11.5	9.45	2.31
Times of UCCS, per day	5	5	5	5	3	5	5	5	4.75	0.71
Lactose-free formula	+	−	+	−	−	+	+	−		
Polymer formula	−	−	+	−	−	+	+	−		
Amino acid formula	+	+	−	+	+	−	−	+		
Mesalazine	+	+	+	+	+	−	+	+		
G-CSF*	+	+	+	+	+	+	+	+		
Glucocorticoid	−	−	−	−	+	−	−	+		
Infliximab	−	+	+	+	−	−	−	−		
Metronidazole	+	+	+	+	+	+	+	+		
Vancomycin	−	−	−	+	−	−	+	+		

UCCS: uncooked corn starch; G-CSF: granulocyte colony-stimulating factor.

At present, dietary management is the first-line treatment for GSD; this treatment maintains blood glucose levels and prevents hypoglycaemia by regular consuming of UCCS, as well as lactose-free formulas, polymer formulas, and elemental formulas. These patients consumed 5.3 to 11.9 g/kg UCCS daily for 3 to 5 meals. Multiple colonic strictures were confirmed by abdominal CT in 4 patients who were unable to eat solid food and who received nasogastric tube feeding. All 8 patients received intermittent G-CSF therapy for neutropenia. Three patients (patients 2, 3, and 4) received three doses of infliximab (5–10 mg/kg) at week 0, 2, and 6, respectively, and no significant improvement in mucosal ulcers or inflammatory hyperplasia was observed via endoscopy at week 14. Six patients received mesalazine (6/8), and two patients received glucocorticoids (2/8) for GSD-associated IBD. Metronidazole and vancomycin were used to treat oral ulcers (8/8) and colitis (3/8), respectively (Table 1).

### Changes in clinical symptoms

All eight patients had common clinical features of GSDs before empagliflozin treatment, including hypoglycaemia (8/8), lactic acidosis (8/8), anaemia (8/8), hepatosplenomegaly (8/8), fatigue (7/8), weakness (7/8), hyperuricaemia (5/8), and hyperlipidaemia (4/8). Gastrointestinal symptoms included abdominal pain (8/8), recurrent oral ulcers (8/8), anorexia (7/8), diarrhoea and/or bloody stool (7/8), nausea/vomiting (6/8), abdominal bloating (6/8), and perianal abscesses (6/8). Other complications of infection were otitis media (2/8) or urinary tract infection (2/8) (Table 3). The clinical symptoms of hypoglycaemia, lactic acidosis, fatigue and weakness, anorexia, abdominal pain, diarrhoea, and/or bloody stool were significantly improved after empagliflozin treatment for 48 weeks. The number of patients with these symptoms decreased by 50%. Recurrent oral ulcers (8/8), anaemia (8/8), and hepatomegaly (8/8) were the three main persistent symptoms in patients with GSD-associated IBD (Table 2).

**Table 2:** Common features and gastrointestinal symptoms.

		Patient 1	Patient 2	Patient 3	Patient 4	Patient 5	Patient 6	Patient 7	Patient 8	N(%)
Hypoglycaemia	Before EMP	+	+	+	+	+	+	+	+	8 (100)
	After EMP	+	−	−	−	−	−	+	+	3 (37.5)
Hepatomegaly	Before EMP	+	+	+	+	+	+	+	+	8 (100)
	After EMP	+	+	+	+	+	+	+	+	8 (100)
Lactic acidosis	Before EMP	+	+	+	+	+	+	+	+	8 (100)
	After EMP	+	−	−	−	−	−	−	+	2 (25.0)
Hyperlipidaemia	Before EMP	−	+	+	+	−	−	+	−	4 (50.0)
	After EMP	−	−	+	+	−	−	+	−	3 (37.5)
Hyperuricaemia	Before EMP	−	−	+	+	+	+	−	+	5 (62.5)
	After EMP	−	−	+	−	+	−	−	−	2 (25.0)
Anaemia	Before EMP	+	+	+	+	+	+	+	+	8 (100)
	After EMP	+	−	+	+	−	+	−	+	5 (62.5)
Fatigue and weakness	Before EMP	+	+	−	+	−	+	+	+	6 (75.0)
	After EMP	−	−	−	−	−	−	+	+	2 (25.0)
Perianal abscess	Before EMP	+	−	+	+	+	+	+	+	7 (87.5)
	After EMP	+	−	−	+	−	−	+	+	4 (50.0)
Oral ulcer	Before EMP	+	+	+	+	+	+	+	+	8 (100)
	After EMP	+	+	+	+	+	+	+	+	8 (100)
Otitis media	Before EMP	+	−	−	−	+	−	−	+	3 (37.5)
	After EMP	−	−	−	−	+	−	−	−	1 (12.5)
Urinary tract infection	Before EMP	−	−	−	+	−	−	−	+	2 (25.0)
	After EMP	−	−	−	−	−	−	−	+	1 (12.5)
Nausea/vomiting	Before EMP	+	−	+	+	+	+	−	+	6 (75.0)
	After EMP	+	−	−	−	−	−	+	+	3 (37.5)
Anorexia	Before EMP	+	+	+	+	+	+	−	+	7 (87.5)
	After EMP	−	−	−	−	−	−	−	+	1 (12.5)
Abdominal pain	Before EMP	+	+	+	+	+	+	+	+	8 (100)
	After EMP	+	−	−	−	−	+	+	+	4 (50.0)
Abdominal bloating	Before EMP	+	+	−	+	−	+	+	+	6 (75.0)
	After EMP	−	−	−	−	−	+	+	+	3 (37.5)
Diarrhoea/bloody stool	Before EMP	+	−	+	+	+	+	+	+	7 (87.5)
	After EMP	−	−	−	−	−	+	−	+	2 (25.0)
Total number of symptom items	Before EMP	13	10	12	15	12	13	11	15	101 (78.9)
	After EMP	8	2	5	5	4	6	9	13	52 (40.6)

EMP: Empagliflozin.

### Changes in laboratory indicators

Compared with those at baseline and before treatment, the blood glucose and lactate levels were more stable after 48 weeks of empagliflozin treatment, and the ANC, hs-CRP, haemoglobin, and triglyceride levels improved, with no statistical difference, while the ESR, platelet count, albumin concentration, and uric acid concentration did not change (Table 3, Fig. 1B).

**Table 3:** Changes in laboratory parameters and adverse events from baseline to week 48.

		Patient 1	Patient 2	Patient 3	Patient 4	Patient 5	Patient 6	Patient 7	Patient 8	Mean	SD	P valure
White blood cell count (10^9^/L)	Baseline	2.9	11.9	3.60	12.5	9.0	2.60	5.40	3.25	6.39	4.13	0.078
	Before EMP	3.0	1.60	3.50	3.90	3.9	3.28	3.66	2.33	3.15	0.81	
	After EMP	2.69	2.25	1.75	3.61	4.07	2.45	2.85	3.28	2.87	0.76	
Neutrophil count (10^9^/L)	Baseline	0.78	1.68	0.61	1.03	0.27	1.52	1.20	1.45	1.07	0.49	0.159
	Before EMP	0.70	0.72	0.88	0.71	0.35	0.73	0.67	0.26	0.63	0.21	
	After EMP	0.88	0.88	0.43	0.38	1.50	0.60	1.54	0.94	0.89	0.44	
Haemoglobin (g/L)	Baseline	76	94	91	87	90	93	99	61	86.38	12.23	0.446
	Before EMP	88	74	84	84	113	83	81	81	83.13	4.85	
	After EMP	59	114	82	84	121	96	115	93	94.50	19.55	
Platelet count (10^9^/L)	Baseline	421	501	623	477	564	619	413	336	494.25	103.07	0.889
	Before EMP	430	421	623	617	405	615	355	311	472.13	126.91	
	After EMP	426	467	466	573	380	469	307	687	471.88	116.07	
hs-CRP (mg/L)	Baseline	79.6	68.4	45.0	60.0	105.4	70.8	200	84	89.15	48.16	0.008
	Before EMP	84.8	167.2	191.7	37.9	27.22	85.5	45.0	88.6	90.99	59.79	
	After EMP	26.7	36.7	10.9	3.7	2.0	72.5	6.0	73.2	28.96	29.61	
ESR (mm/1 h)	Baseline	49	93	120	20	99	45	60	68	69.25	32.81	0.231
	Before EMP	48	90	117	19	68	76	41	119	72.25	35.73	
	After EMP	37	89	56	26	24	60	23	115	53.75	33.64	
Blood glucose (g/L)	Baseline	5.90	6.13	8.14	7.35	4.40	4.20	5.42	2.40	5.49	1.83	0.688
	Before EMP	9.03	6.13	2.72	2.8	3.90	4.50	4.0	4.86	4.74	2.05	
	After EMP	4.82	5.48	5.13	4.7	5.67	4.45	6.50	4.85	5.20	0.66	
Lactate (mmol/L)	Baseline	1.50	19.60	5.20	15.00	11.8	3.10	2.50	2.18	7.61	6.92	0.101
	Before EMP	2.36	5.90	6.50	17.10	9.80	2.78	8.30	1.10	6.77	5.25	
	After EMP	3.78	2.35	2.71	3.88	3.50	1.97	2.71	3.3	2.91	0.68	
Albumin (g/L)	Baseline	24.6	43.6	45.3	40.0	26.8	27.6	26.2	26.5	32.58	8.77	0.419
	Before EMP	25.7	35.5	33.9	37.1	34.4	31.5	26.2	29.8	31.14	4.35	
	After EMP	29.8	36.0	37.9	36.6	40.8	31.6	39.1	27.4	34.9	4.76	
Uric acid (umol/L)	Baseline	340	383	604	256	427	397	423	358	398.5	99.47	0.159
	Before EMP	340	299	660	609	546	558	279	478	471.13	147.17	
	After EMP	254	239	533	249	577	473	317	239	360.13	143.66	
Cholesterol (mmol/L)	Baseline	3.89	3.70	5.85	7.26	2.64	4.47	5.18	2.45	4.43	1.63	0.686
	Before EMP	4.56	3.32	2.77	6.04	5.28	4.71	6.22	2.96	4.48	1.35	
	After EMP	3.79	4.90	3.08	4.94	1.83	5.40	4.53	3.71	4.02	1.17	
Triglyceride (mmol/L)	Baseline	1.71	2.02	6.63	11.48	2.12	1.16	2.08	2.13	3.67	3.58	0.417
	Before EMP	1.53	5.65	2.52	5.76	3.5	1.73	8.72	1.91	3.92	2.57	
	After EMP	1.79	1.83	3.19	3.59	1.0	1.26	2.23	1.10	1.99	0.96	
PCDAI	Baseline	80	80	67.5	70	75	65	60	85	72.81	8.60	0.001
	Before EMP	50	50	45	40	35	47.5	45	65	47.19	8.81	
	After EMP	37.5	20	22.5	22.5	20	37.5	42.5	55	32.19	12.92	
wPCDAI	Baseline	107.5	100	80	85	97.5	87.5	80	117.5	94.375	13.61	0.000
	Before EMP	62.5	67.5	60	42.5	70	65	62.5	80	63.75	10.61	
	After EMP	42.5	32.5	32.5	20	25	47.5	62.5	70	41.5625	17.67	
SES-CD	Before EMP	23	8	18	18	4	21	6	16	14.25	7.23	0.045
	After EMP	13	2	2	4	2	11	7	17	7.25	5.80	
Adverse events												
Grade 3 hypoglycemia episode /year	Before EMP	5	2	2	3	4	0	5	4	3.13	1.73	0.003
	After EMP	1	1	0	1	2	0	2	2	1.13	0.83	
Sweating	After EMP	Negative	Negative	Negative	Negative	Negative	Negative	Negative	Positive			
Urinary tract infection	After EMP	Negative	Negative	Negative	Negative	Negative	Negative	Negative	Positive			

EMP: Empagliflozin; ESR: Erythrocyte sedimentation rate; PCDAI: Paediatric Crohn’s disease activity index; SES-CD: Simple Endoscopic Scale for Crohn’s Disease.

### Changes in the PCDAI score

The PCDAI score decreased significantly at week 48 (32.19 ± 12.92) compared with that at baseline (72.81 ± 8.60) and before empagliflozin (47.19 ± 8.81) (P < 0.001 and P = 0.005, respectively) (Table 3 and Fig. 1C). In addition, the PCDAI score decreased by ≥ 12.5 points in 5 of the 6 patients who were regularly administered empagliflozin for 48 weeks, for a clinical response rate of 83.3% (5/6). Among them, 4 patients had PCDAI scores of ≤ 30 points (patients 2, 3, 4, and 5), indicating moderate/severe disease improvement to mild disease activity. Due to the specific growth failure of GSD-associated IBD, we compared the wPCDAI score^23^ with the PCDAI score. After 48 weeks of treatment with empagliflozin, the wPCDAI score was severe in 2 cases (2/8, 25%) and the PCDAI score was severe in 4 cases (4/8, 50%). The moderate improvement in the PCDAI score in 2 cases (25%), wPCDAI score was in one case (12.5%). Both scoring systems were consistent in terms of moderate scoring and small improvement, but the wPCDAI score was easier to use.

### Endoscopic features and histological activity

**Figure 2:**
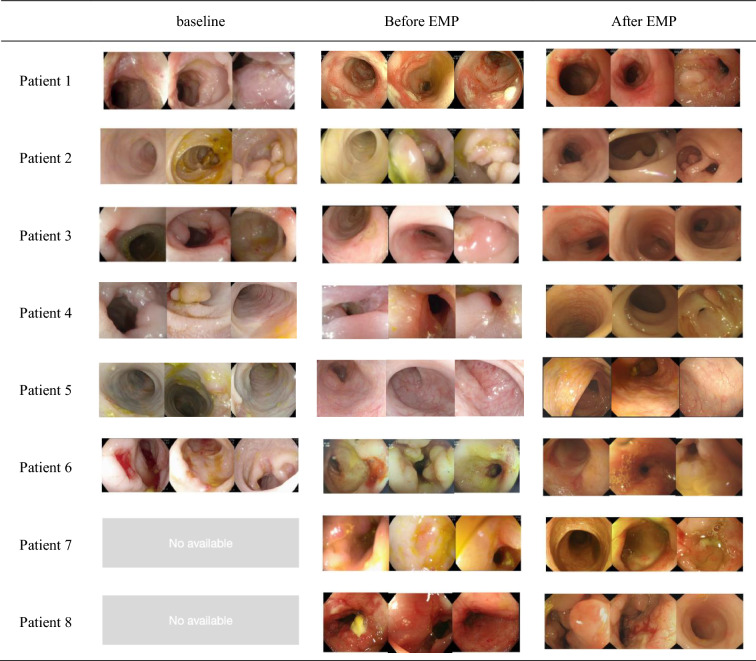
Endoscopic image from baseline to week 48. The colonic mucosa shows edema, loss of vascular network, scattered erosion, deep large circular ulcers, inflammatory hyperplasia, obstruction, and stenosis. Before treatment with empagliflozin , 5 of the 8 patients had colonic stenosis (patients 3,4,6,7, and 8) and one had colonic obstruction (patient 2). The stenosis sites were ascending colon (patient 3), transverse colon (patients 2 and 4), and descending colon (patients 6,7, and 8), respectively. After treatment with empagliflozin, colon stenosis and obstruction were significantly improved (patients 2 and 4) or disappeared (patient 3), and colon stenosis occurred in the ascending (patient 4), transverse (patient 1), and descending (patients 6, 7, and 8). *EMP: empagliflozin.

**Figure 3:**
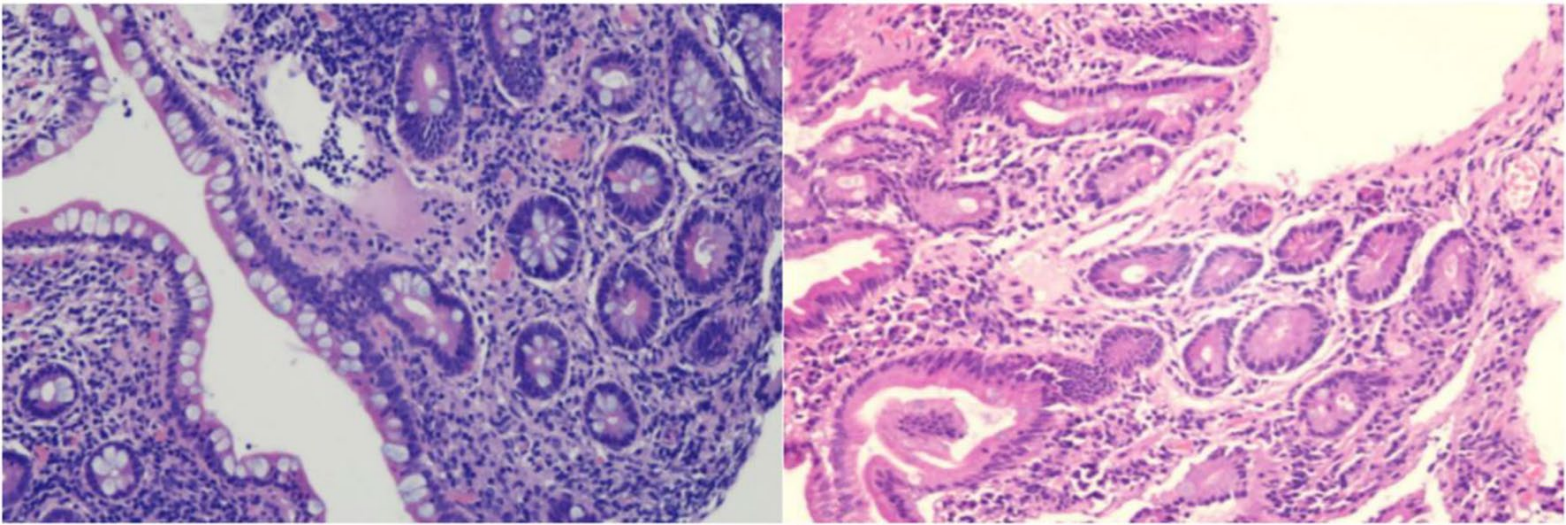
Histology finding in GSD-associated IBD. Chronic inflammatory infiltration of the mucous lamina propria, marked increase in lymphocytes and plasma cells (grade 1.3), or mild but definite increase in eosinophils (1–14/HPF) (grade 2A.1), no neutrophil infiltration in the epithelium and lamina propria, and no granuloma or crypt abscess observed in GSD-associated IBD according to Geboes methods.

The colonic mucosa of all patients showed obvious congestion, oedema, and erosion, and the vascular network disappeared before the application of empagliflozin. Seven patients had deep and large circular ulcers, and six patients had colonic obstruction and stenosis. Unlike the features of classical IBD, there were no longitudinal ulcers or cobblestone-like mucosal lesions (Fig. 2). Colonic obstruction and stenosis were present in 6 of the 8 patients on the first endoscopy and before empagliflozin treatment, and 4 cases had colonic stenosis after treatment, and the diameter of 5 mm endoscope could not pass through the stenosis site. Except for no significant improvement in patients 6, 7, and 8, the obstruction and stenosis disappeared after 48 weeks in patients 2 and 3, and the stenosis in patient 4 was less than 1 cm in length at the proximal ileum of the ascending colon. The esophagogastroduodenoscopy (EGD) revealed mild chronic inflammation in the antral or duodenal bulb at baseline, without ulcer or inflammatory hyperplasia, which resolved after PPI therapy. The EGD and colonoscopy showed no mucosal ulceration and hyperplasia of the duodenum and distal ileum before empagliflozin treatment (the endoscopic images were not uploaded). The patients did not undergo capsule endoscopy due to the high incidence of colonic stenosis in patients with GSD-associated IBD. The SES-CD score significantly decreased at 48 weeks (7.25 ± 5.80) compared with before empagliflozin treatment (14.25 ± 7.25), (P = 0.046) (Table 3 and Fig. 1D). 5 patients who were regularly administered empagliflozin for 48 weeks achieved significant improvements, including mucosal ulcer repair (4/4), a reduction in inflammatory hyperplasia (3/3), and a reduction in obstruction and stenosis (3/3), as assessed by endoscopy, while no significant improvement was observed in one patient who received regularly for 48 weeks (patients 7) and 2 patients (patients 6 and 8) who were treated irregularly no more than 8 weeks (Fig. 3). The Geboes method, a widely used scoring index, has shown good reproducibility^27^. The pathological assessment of colonic mucosa in this group of patients by the Geboes method revealed chronic inflammatory infiltrates with marked increases in lymphocytes and plasma cells (grade 1.3) or mild but unequivocal increases in eosinophils (1–14/HPF) in the lamina propria of the mucosa (grade 2A.1); no neutrophil infiltration in the epithelium or lamina propria; and no granulomas or crypt abscesses (Fig. 3).

### Adverse events during empagliflozin use

An adverse event is defined as any adverse or unexpected event observed after the administration of a pharmaceutical product. Grade 3 hypoglycemia is a common serious complication of GSD-Ib. In this study, a total of 9 episodes of hypoglycaemia occurred in 8 patients, which was significantly less than the 25 episodes in the first 12 months before empagliflozin (P = 0.003) (Table 3). One patient (patient 8) started the drug and experienced severe sweating, needed rehydration, developed a urinary tract infection, and subsequently abandoned the empagliflozin treatment after two attempts with the same symptoms. In this group, there were no new safety issues, life threats or deaths.

## Discussion

Here, we described eight paediatric patients diagnosed with GSD-Ib by genetic testing in the first year of life and with GSD-associated IBD by endoscopy at the age of 7.64 years (range 2.6–9.8 years). We also summarized the common features of GSDs and gastrointestinal symptoms in children with GSD-associated IBD. In addition to neutropenia, all eight of the patients presented with hypoglycaemia, hepatomegaly, lactic acidosis, and anaemia; 6 patients presented with fatigue and weakness; and 4 patients had hyperlipidaemia or hyperuricaemia. Gastrointestinal symptoms included abdominal pain, recurrent oral ulcers, anorexia, diarrhoea and/or bloody stool, nausea/vomiting, abdominal bloating, and perianal abscess. Recurrent oral ulcers, anaemia, and hepatomegaly were the three persistent symptoms in patients with GSD-associated IBD. Dietary management is the first-line treatment for GSDs to maintain blood sugar and prevent hypoglycaemia, with regular use of UCCS. Eight patients in this group consumed UCCS at a dose of 9.45 g/kg/day (range 5.3–11.9 g/kg/day) at enrolment, and their parents complained of diarrhoea and abdominal pain after starting UCCS. A 5-year-old boy (patient 5) who had been continuously fed an elemental formula through a nasogastric tube for more than 3 years developed diarrhoea, bloating, and mouth ulcers after repeated UCCS restarts. IBD is an abnormal immune response triggered by environmental factors on a genetic basis. Most researchers believe that neutropenia and neutrophil dysfunction are the main causes of IBD in patients with GSD-Ib^28^. However, IBD has been reported in GSD-Ia^29^ and GSD-III^30^ patients with normal neutrophils. Whether UCCS is one of the causes of digestive symptoms and IBD needs further study.

Patients in this cohort were enrolled at 10.34 years of age (4.5 to 12.5 years), nearly 3 years after their first endoscopy, and nearly 10 years after diagnosis with GSD-Ib. All eight patients had previously received G-CSF treatment for neutropenia. Compared with the first endoscopic images, there was no significant effect on the prevention or treatment of IBD, probably because G-CSF can improve the neutrophil count but not the neutrophil dysfunction^14^. Similarly, glucocorticoids and infliximab have not been shown to alleviate colonic mucosal ulceration, inflammatory hyperplasia, or stenosis in GSD-associated IBD patients.

Recently, after Wortmann et al. reported the use of empagliflozin for treating neutropenia and neutrophil dysfunction in GSD-Ib patients^18^, more than 230 adult or paediatric patients with GSD-Ib who received empagliflozin have been reported in more than 20 pieces of literature, including two international questionnaire studies^20,31^ and some case reports^21,32–36^. The efficacy in these studies was assessed by clinical symptoms and the PCDAI ^6,28^, empagliflozin has been recommended for the treatment of neutrophil dysfunction in GDS-Ib patients with or without IBD. We found that, before treatment with empagliflozin, patients with GSD-Ib exhibited colonic mucosal oedema and erosion; their vascular network disappeared; and they had distinctive deep and large circular ulcers, obstructions, and stenoses, unlike in classical IBD, in which no longitudinal ulcers or cobblestone-like mucosal lesions were observed via endoscopy; such cases were diagnosed as GSD-associated IBD. Six patients had colonic obstruction and stenosis. The EGD revealed mild chronic inflammation in the antral and/or duodenal bulb at baseline, which resolved with PPI therapy before empagliflozin treatment. The EGD and colonoscopy showed no ulcers and hyperplasia in the mucosa of the duodenum and ileum. Ultrasound and abdominal CT scan showed no lesions in the small intestine. Thus, the clinical challenge in GSD-associated IBD is colonic lesions, especially colonic deep ulcers, stenosis, and obstruction. The pathological examination showed chronic inflammation, Geboes grade 1.3 to grade 2A.1, no neutrophil infiltration, and no granulomas or crypt abscesses. In this study, the overall response rate of empagliflozin was 62.5% (5/8). Six patients were treated with 0.25–0.50 mg/kg/d empagliflozin regularly, and the clinical effective rate was 83.3% (5/6) after 48 weeks, with the PCDAI score decreasing by > 12.5 points in 5 patients and by < 30 points in 4 patients. Since there were only 8 patients in this group, the PCDAI results were consistent with the wPCDAI, but wPCDAI easier to perform. The colonic mucosal ulcers were relieved, inflammatory hyperplasia was observed, and colonic stenosis was observed; moreover, the SES-CD score was significantly improved or eliminated by endoscopy. One patient (patient 7) received empagliflozin with 0.27 mg/kg/d regularly for 48 weeks, and two patients (patients 6 and 8) received 0.20–0.22 mg/kg/d irregularly for no more than 8 weeks, the PCDAI score was 37.5–55, and there was no improvement in colonic mucosal lesions, moreover, the degree and extent of colonic stenosis increased after 48 weeks. Therefore, the reasons for the ineffectiveness of the 3 patients may be: irregular drug use led to low blood drug concentration, insufficient maintenance of treatment time, and possible differences in individual pharmacodynamics and pharmacokinetics.

Episodes of grade 3 hypoglycemia, a GSD-Ib clinical crisis, did not increase in this study with empagliflozin treatment, and on the contrary, the number of such episodes decreased significantly after treatment with empagliflozin. One patient reported discontinuation of medication due to excessive sweating requiring rehydration, as well as a urinary tract infection. No serious adverse events were reported, no new safety concerns were reported ^31^, and no life-threatening conditions or deaths occurred.

Our study has several limitations. First, evaluating disease activity in GSD-associated IBD patients is a clinical challenge. Clinical symptoms such as diarrhoea and anorexia in children with GSD-Ib may improve with age, and colonic mucosal ulcers, inflammatory hyperplasia, obstruction, and stenosis may not be alleviated with age and may even be aggravated; thus, the PCDAI score has limitations. Neutrophil infiltration in the epithelium at different sites may be affected by neutropenia, which may affect the Geboes score. Among the 8 patients in this group, 5 cases had colonic stenosis and one had colonic obstruction before treatment, and 4 cases had colonic stenosis after treatment, and the diameter of 5 mm endoscope could not pass through the stenosis site. Except for no significant improvement in patients 6, 7, and 8, the obstruction and stenosis disappeared in patients 2 and 3, and the stenosis in patient 4 was less than 1 cm in length at the proximal ileum of the ascending colon. The significant reduction in the SES-CD score after 48 weeks of treatment was based on three patients (patient 2, 3, and 4) who had significant improvement or resolution of stenosis and obstruction after treatment. Due to the high incidence and severity of colonic stenosis in this group, and the inability to evaluate the colon beyond the stenosis site, the SES-CD score has certain limitations for GSD-associated IBD. Second, although empagliflozin seems to have clear benefits for patients with GSD-associated IBD, there are still many unanswered questions. When does it start? What are the clear indications? What is the optimal dose and duration? What are the individual pharmacogenetic and pharmacokinetic factors that influence the therapeutic effect? Are there any other side effects?

In conclusion, this study comprehensively evaluated the efficacy of empagliflozin in terms of clinical symptoms, laboratory indices, and PCDAI scores, especially endoscopic features, the SES-CD score, and the Geboes score. After 48 weeks of empagliflozin treatment, patients showed significant improvement in clinical symptoms and mucosal lesions.

These findings provide strong evidence and experience for the use of empagliflozin in the treatment of GSD-associated IBD. Future research needs to address the problem: the indications, contraindications, dosage, duration, the factors affecting individual pharmacodynamics and pharmacokinetics, and other drug mechanisms other than neutrophils as targets of empagliflozin.

## Data Availability

All data described in this study are provided within the article and supplementary materials. Additional de-identified clinical data are available upon http://www.medresman.org.cn.
